# Prevalence, comorbidity and predictors of social anxiety severity among Chinese youth in the post-COVID-19 era

**DOI:** 10.1192/bjo.2026.10980

**Published:** 2026-02-26

**Authors:** Yi-Zhou Wang, Khalid Imran Afzal, Cheng-Mei Yuan, Jing-Yi Fan, Jun He, Xu-Hong Li, Bei-Bei Wang, Yu-Ya Feng, Hong-Yun Shao, De-Hui Zhou, Xue Weng

**Affiliations:** Department of Counselling and Psychology, Hong Kong Shue Yan University, North Point, Hong Kong SAR, China; Department of Psychiatry and Behavioral Neuroscience, The University of Chicago Medicine, Chicago, Illinois, USA; Clinical Research Center, Shanghai Mental Health Center, Shanghai Jiao Tong University School of Medicine, Xuhui, Shanghai, China; Department of Paediatrics, Zhongnan Hospital of Wuhan University, Wuhan, Hubei, China; Department of Social and Behavioural Sciences, City University of Hong Kong, Kowloon, Hong Kong SAR, China; School of Education, Xin Yang University, Xinyang, Henan, China; Centre of Psychological Health Education, Jiangsu University of Science and Technology, Suzhou, Jiangsu, China; Beijing Institute of Education, Tongzhou, Beijing, China; Institute of Advanced Studies in Humanities and Social Sciences, Beijing Normal University, Zhuhai, Guangdong, China

**Keywords:** Social anxiety, youth, depression, stigma, avoidance behaviour

## Abstract

**Background:**

Social anxiety is a common and impairing condition that often emerges in adolescence.

**Aims:**

This study aimed to examine the prevalence and severity of social anxiety among Chinese youths in the post-COVID-19 era, and to develop a predictive model identifying key factors associated with social anxiety severity.

**Method:**

A total of 555 youths aged 15–25 years completed an online survey via WeChat on social anxiety (Social Phobia Inventory), depressive symptoms (Patient Health Questionnaire), sleep problems (Pittsburgh Sleep Quality Index), social support (Multidimensional Scale of Perceived Social Support) and internalised stigma (Internalized Stigma of Mental Illness Scale). Social anxiety severity and rates were described, and comparisons were made across sociodemographic groups. Hierarchical multiple regression was used to predict social anxiety severity from depression, sleep, social support and stigma. An additional regression examined which components of social anxiety (fear, avoidance, physical symptoms) predict internalised stigma.

**Results:**

In total, 69.55% of participants reported at least mild social anxiety, with 20% reaching severe or very severe levels. Female, younger participants and those with fewer close friends reported significantly higher anxiety. Depressive symptoms (*β* = 0.31, *P* < 0.05) and internalised stigma (*β* = 0.40, *P* < 0.05) were strong predictors of anxiety severity, while sleep problems and social support were not significant after controlling for these factors. Among social anxiety dimensions, only avoidance significantly predicted higher stigma (*β* = 0.17, *P* < 0.01).

**Conclusions:**

The high post-pandemic prevalence of social anxiety among youths highlights the need for early identification, stigma reduction and interventions targeting depression and avoidance to prevent long-term impairments.

## Definition and impacts of social anxiety

Social anxiety is characterised by intense fear of social or performance situations and worry about scrutiny or embarrassment.^1^ It typically has an onset in adolescence and is among the most prevalent anxiety disorders in youth, defined by the World Health Organization (WHO) as those aged 15–24 years.^2^ Across a global sample of 6825 individuals from countries including China, the USA, Indonesia, Russia, Thailand, Brazil and Vietnam, the prevalence of social anxiety was found to be significantly higher than previously reported. Notably, over one in three respondents (36%) met the threshold criteria for social anxiety disorder (SAD).^3^ Although social anxiety is distinct from a clinical diagnosis of SAD, untreated symptoms can progressively worsen, leading to significant impairments in peer relationships and academic performance and, in some cases, school refusal.^4^ Chronic school refusal, defined as persistent difficulty attending school, is associated with long-term academic and social dysfunction. Youths with social anxiety who develop school refusal frequently show limited response to interventions and continued functional impairment.^5^ In such cases, even intensive therapeutic efforts may not fully reintegrate the adolescent into regular schooling. These findings underscore the importance of early identification and intervention for social anxiety, to prevent lasting educational and psychosocial consequences.

## Comorbidity of social anxiety

Social anxiety in youths frequently co-occurs with depression.^6^ Longitudinal studies suggest a reciprocal relationship, where early social anxiety can increase the risk for later depression.^7^ Emotional patterns in social anxiety comprise a cyclical surge of fear, anticipatory tension and behavioural avoidance, whereas cognitive patterns entail maladaptive appraisals and beliefs that govern the anticipation, interpretation and post-event evaluation of social interactions.^8^ Acting synergistically, these emotional and cognitive processes can precipitate depressive symptoms and, once depression emerges, with its anergia, anhedonia and reduced self-efficacy, it further amplifies social fears.^9^

Given their frequent comorbidity and mutual reinforcement, examination of depressive symptoms alongside social anxiety severity is crucial. In addition to depression, internalised stigma may also play a key role in shaping social anxiety severity. Youths with social anxiety often report experiencing stigma – feeling judged, inferior or socially undesirable because of their mental health challenges.^10^ This internalised stigma may exacerbate social anxiety by reinforcing fears of negative evaluation. For instance, if a teenager believes that others will view them as ‘weird’ or ‘weak’ for being anxious, they may become increasingly avoidant in social situations. In turn, heightened avoidance may deepen their sense of being stigmatised, creating a self-perpetuating cycle of isolation and fear.^10^ While these theoretical links are well documented, empirical research specifically examining the role of stigma in youth social anxiety is limited, and its mechanisms remain poorly understood.

Beyond depression and stigma, sleep problems are common among anxious individuals, and poor sleep may heighten anxiety through mechanisms such as cognitive rumination and physiological hyperarousal.^11^ Likewise, social support is generally viewed as a protective factor, whereas loneliness or a lack of perceived support may increase vulnerability to emotional distress.^12^ Socially anxious youths often have fewer close connections and are more likely to withdraw from social interactions, which may both result from and reinforce their anxiety.^13^ However, it remains unclear how these factors jointly operate within a single predictive model for post-COVID Chinese youth.^6^

## Post-pandemic impacts on social anxiety

Post-COVID-19 pandemic shifts in daily life have been linked to social avoidance and anxiety among adolescents. Prolonged periods of remote learning, mask mandates and social distancing mean that youths had far fewer face-to-face interactions during their critical developmental years, and this enforced social isolation has been associated with increases in anxiety and other mental health problems in young people.^14^ The normalisation of mask-wearing, while protective from a public health perspective, inadvertently provided socially anxious youths with a ‘safety shield’ – allowing them to hide facial expressions or perceived imperfections – which can reinforce avoidant coping and make the prospect of fully unmasked social interaction more intimidating.^15^ Many teenagers also grew accustomed to avoiding public spaces and in-person gatherings (the pandemic ‘new normal’), and thus missed opportunities to practise social skills; as a result, some now experience intense anxiety about re-engaging in ordinary social or school activities.^14^ Studies report that youths often feel vulnerable and anxious without pandemic precautions (like masks) in place, suggesting that the transition back to regular social life has unmasked a residual reluctance and heightened social anxiety in the post-pandemic era.^15,16^

In light of the above, this study aims to: (a) examine the prevalence and severity of social anxiety among Chinese youths in the post-COVID-19 context (objective 1); and (b) develop an integrated predictive model that incorporates key psychological and social factors, including depression, sleep quality, social support and stigma (objective 2).

## Method

### Participants

Participants aged 15–25 years were recruited for a cross-sectional online survey on youth mental health, conducted from September to December 2024. The age range was chosen to align with the WHO definition of youth (15–24 years) and to include early-young adults (25 years), to capture transitional experiences post-high school. Inclusion criteria also included the ability to complete the Chinese survey and the provision of informed consent (parental consent for those under 18 years). Exclusion criteria were incomplete responses and ages outside the specified range. A total of 555 participants were drawn from both high school and university settings and via online outreach, to include older adolescents and young adults. Inclusion criteria required that participants be within the target age range and provide informed consent (for minors, parental consent and youth assent were obtained). Surveys were completed anonymously online via a secure WeChat Mini platform (OkeyMind).

### Ethical standards

Ethical approval was granted by the Human Research Ethics Committee of Hong Kong Shue Yan University (no. HREC 24-04^6^). Each eligible participant willing to join the study signed an online consent form and received compensation of 50 Chinese yuan (approximately £6) for their participation. To ensure the confidentiality and security of participant data, all collected information was securely encrypted and was limited to authorised research personnel during transmission and storage. The study used de-identified or anonymised personally identifiable information data, to safeguard participant privacy wherever possible.

### Measurements

Social anxiety was assessed using the 17-item Social Phobia Inventory (SPIN), a widely used self-report measure rated on a 5-point scale (0–4), with total scores ranging from 0 to 68. Higher scores indicate greater social anxiety severity. SPIN has three established subscales assessing key dimensions of social anxiety: fear (e.g. fear of embarrassment or scrutiny), avoidance (avoidance of social situations) and physiological symptoms (physical manifestations of anxiety, such as blushing or sweating).^17^ The Chinese version of SPIN has been examined and was found to have good internal consistency (Cronbach’s *α* = 0.85) and test–retest reliability (*r* = 0.73).^18^ In this study, SPIN scores were also categorised to describe severity: typically, scores <21 are considered non-clinical, 21–30 indicate mild social anxiety, 31–40 moderate, 41–50 severe and 51–68 very severe.^17^ These cut-offs were used to classify participants into severity groups for descriptive analyses.

Depressive symptoms were measured using the Patient Health Questionnaire (PHQ-9), a widely used 9-item self-report scale assessing depression severity. Items are rated from 0 (not at all) to 3 (nearly every day), yielding a total score of 0–27. Depressive symptom severity was categorised as follows: <5, none; 5–9, mild; 10–14, moderate; 15–19, moderately severe; and ≥20, severe. The Chinese version of PHQ-9 demonstrates high internal validity and consistency, evidenced by a Cronbach’s *α* of 0.86, strong test–retest reliability and a correlation coefficient of 0.86, affirming its efficacy for use in Chinese-speaking populations.^19^

Sleep quality was measured using the 19-item Pittsburgh Sleep Quality Index (PSQI), which assesses sleep disturbances and overall sleep quality over the past month. PSQI yields a global score ranging from 0 to 21, with scores >5 indicating poor sleep quality. The Chinese version of PSQI has demonstrated good test–retest reliability (*r* = 0.85), supporting its validity in Chinese-speaking populations.^20^

Social support was measured in two ways. First, participants reported the number of close friends they could rely on (categorised as 0–3, 4–6 or ≥7 friends) as an objective indicator of their social network size. Additionally, social support was assessed using the 12-item Multidimensional Scale of Perceived Social Support (MSPSS), which evaluates the perceived adequacy of support from three sources: family, friends and a significant other. Each item is rated on a 7-point Likert scale ranging from 1 (very strongly disagree) to 7 (very strongly agree), with higher scores indicating greater perceived support. The Chinese version of MSPSS has demonstrated strong psychometric properties, with an internal consistency coefficient of 0.911, a split-half reliability of 0.865 and test–retest reliability ranging from 0.837 to 0.914.^21^

Stigma was assessed using the 10-item short form of the Internalized Stigma of Mental Illness Scale (ISMI-10), which evaluates the extent to which individuals internalise negative beliefs and stereotypes related to mental illness. Each item is rated on a 4-point Likert scale ranging from 1 (strongly disagree) to 4 (strongly agree), with higher scores indicating greater internalised stigma.^22^

### Data analysis

All analyses were conducted using SAS version 9.4 for Windows (SAS Institute Inc., Cary, North Carolina, USA; https://www.sas.com). To address objective 1, group comparisons were performed to examine differences in social anxiety scores across sociodemographic variables. Independent-samples *t*-tests were used for binary variables (e.g. female versus male, non-smoker versus smoker), and one-way analysis of variance was applied to multi-category variables (e.g. age group, income level). Confounders (gender, age, smoking status and number of close friends) were selected based on significant associations (see Table 1) following multiple-testing adjustment), and were included as control variables in all regression models. Social anxiety severity was classified according to standard SPIN cut-offs^17^: non-clinical (<21), mild (21–30), moderate (31–40), severe (41–50) and very severe (51–68).

**Table 1:** Sociodemographic characteristics and social anxiety of youths (*N* = 555)

Variables	*N*	%	SAD			
			Mean	s.d.	*t/F*-value	*P*-value
Gender					−3.52	<0.01**
Female	434	78.20	29.86	13.12		
Male	121	21.80	24.60	14.93		
Age, years					7.49	<0.01**
<18	51	9.19	35.14	14.25		
18–22	473	85.23	27.83	13.49		
23–25	31	5.59	31.68	13.18		
Family income, RMB (£)					1.79	0.15
≤10 000 (1040)	93	16.76	31.37	13.30		
10 001–50 000 (1040–5200)	152	27.39	29.91	14.04		
50 001–100 000 (5200–10 400)	162	29.19	28.36	13.15		
** **>100 000 (10 400)	148	26.67	27.24	14.07		
Smoking (missing 3)					2.39	0.02*
** **Never	517	93.66	29.10	13.62		
** **Current or previously smoked but quit now	38	6.34	23.53	13.90		
BMI					0.15	0.86
** **<18.5 (underweight)	104	18.74	29.29	12.60		
** **18.5–24.9 (normal weight)	363	65.41	28.67	13.90		
** **≥25 (overweight)	88	15.86	28.22	14.24		
Friends that can be relied on					15.48	<0.01**
** **0–3	381	68.65	30.68	13.76		
** **4–6	139	25.05	25.51	12.38		
** **≥7	35	6.31	20.06	12.51		

SAD, social anxiety disorder; BMI, body mass index. *P < 0.05, **P < 0.01.

For objective 2, a hierarchical multiple regression analysis was conducted to examine predictors of social anxiety severity while controlling for key demographic factors. Predictors (depression, sleep quality, social support, stigma) were selected based on prior literature showing their associations with social anxiety.^6,10–13^ Demographic controls were chosen from significant associations (see Table 1). In the first block, control variables (e.g. gender, age group, smoking status and number of close friends) were entered to account for their potential confounding effects. Subsequent models added psychosocial predictors sequentially: model 1 included depression, model 2 added sleep quality, model 3 introduced perceived social support and model 4 (final model) incorporated stigma. This stepwise approach facilitated assessment of the incremental variance explained by each psychosocial factor beyond the controlled demographic variables. Standardised regression coefficients (*β*), standard errors, *P*-values and model *R* ^2^ values were reported at each step. Analyses were conducted on a subset of participants (*n* = 255) who met the threshold for at least mild social anxiety (SPIN score ≥25). Missing data (approximately three to five cases per variable) were imputed using mean substitution. Statistical significance was set at *P* < 0.05 (two-tailed).

Finally, a separate multiple regression analysis was conducted, with stigma as the outcome variable and the three SPIN subscale scores – fear, avoidance and physical symptoms – as simultaneous predictors. This analysis aimed to identify which dimension of social anxiety was most strongly associated with internalised stigma.

## Results

### Demographic features of social anxiety

This study included 555 participants recruited between September and December 2024. Table 1 presents the descriptive statistics and group comparisons for social anxiety severity across key sociodemographic variables. The sample consisted predominantly of female participants (78.20%), with the majority aged 18–22 years (85.23%). Regarding socioeconomic status, over half of the participants reported a family income of between 10 001 and 100 000 RMB (approximately £1040–10 400). Most participants had never smoked (93.66%), and nearly two-thirds (65.41%) had a normal body mass index (BMI) range of 18.5–24.9. Most (68.65%) reported relying on three or fewer close friends.

Significant differences in social anxiety scores were observed across several demographic variables. Female participants reported significantly higher levels of social anxiety than males (*t* = −3.52, *P* < 0.01). Age group was also significantly associated with anxiety severity, with younger individuals (<18 years) reporting the highest scores (*F* = 7.49, *P* < 0.01). Smoking status was a significant factor, with current or former smokers showing lower social anxiety levels compared with non-smokers (*t* = 2.39, *P* = 0.02). In addition, the number of close friends was strongly associated with anxiety levels: individuals with <4 close friends reported significantly higher social anxiety than those with larger social networks (*F* = 15.48, *P* < 0.01). No significant differences were found for social anxiety by family income or BMI category.

### Severity level of social anxiety in youths

Participants were categorised using standard SPIN cut-offs: <21 (non-clinical, *n* = 169, 30.45%), 21–30 (mild, *n* = 144, 25.95%), 31–40 (moderate, *n* = 131, 23.60%) and 41–50 (severe) and 51–68 (very severe) combined (*n* = 111, 20.00%). Most participants in the study exhibited some degree of social anxiety, with over two-thirds (*n* = 386, 69.55%) reporting at least mild symptoms. Table 2 presents the distribution of social anxiety severity among the youth sample, highlighting a substantial proportion of individuals experiencing clinically relevant symptoms. Among youths presenting with social anxiety symptoms, 50.20% exhibited moderate to severe depressive symptoms.

**Table 2:** Rate and severity of social anxiety in youths (*N* = 555)

Variable	Non-social anxiety		Mild (Social Phobia Inventory (SPIN)) 21–30)		Moderate (SPIN 31–40)		Severe (SPIN 41–50) and very severe (SPIN 51–68)	
	*N*	%	*N*	%	*N*	%	*N*	%
Social anxiety	169	30.45	144	25.95	131	23.60	111	20.00

### Influencing factors of social anxiety in youths

Hierarchical multiple regression analyses (Table 3) were conducted on the full sample (*N* = 555) to examine predictors of social anxiety among young people. To evaluate the robustness of the findings, sensitivity analyses using alternative analytic samples were performed and are presented in the Appendix. These included: (a) participants with SPIN ≥21, representing at least mild symptoms (*n* = 386); and (b) participants with SPIN ≥25, reflecting a more conservative threshold often applied in Chinese populations (*n* = 255).^23^ Across different samples/cut-off scores, *R* ^2^ values were highly comparable (full sample, *R* ^2^ = 0.24; SPIN ≥20, *R* ^2^ = 0.23; SPIN ≥25, *R* ^2^ = 0.24), indicating that the results are stable regardless of sample definition. In the final model, depression remained a significant predictor of social anxiety (*β* = 0.31, s.e. = 0.13, *P* < 0.05), even after accounting for sleep quality, social support and stigma. Stigma was also independently associated with social anxiety severity (*β* = 0.40, s.e. = 0.17, *P* < 0.05). The final model explained 24% of the variance in social anxiety severity (*R* ^2^ = 0.24), suggesting that depression and internalised stigma are the most salient psychosocial correlates of youth social anxiety in this sample. Variance inflation factor diagnostics were conducted to examine potential multicollinearity among PHQ, PSQI and SPIN. The observed variance inflation factor value of 1.68 was below the commonly used threshold of 5, indicating no collinearity concern.^24^

**Table 3:** Hierarchical regression model for all youth samples (*N* = 555)

Variable	Model 1			Model 2			Model 3			Model 4			Model 5		
	*β*	s.e.	*P*-value	*β*	s.e.	*P*-value	*β*	s.e.	*P*-value	*β*	s.e.	*P*-value	*β*	s.e.	*P*-value
Intercept	38.70	5.07	<0.01**	48.68	5.09	<0.01**	47.74	5.11	<0.01**	53.32	6.80	<0.01**	43.36	7.96	<0.01**
Male ref. female	3.75	1.42	<0.01**	−0.82	1.50	0.59	−0.70	1.49	0.64	−0.47	1.50	0.76	−0.30	1.49	0.84
Age	−3.18	1.47	<0.05*	−1.78	1.18	0.14	−1.92	1.18	0.11	−1.83	1.19	0.12	−1.69	1.18	0.15
Smoking	−3.73	2.28	0.10	−4.60	2.33	<0.05*	−4.53	2.33	<0.05*	−5.01	2.35	<0.05*	−4.63	2.34	<0.05*
Friends that can be relied on	−4.67	0.95	<0.01**	−3.48	1.02	<0.01**	−3.28	1.04	<0.01**	−3.02	1.06	<0.01**	−2.89	1.05	<0.01**
Depression				0.53	0.10	<0.01**	0.41	0.12	<0.01**	0.39	0.12	<0.01**	0.31	0.13	<0.05*
Sleeping							0.18	0.11	0.11	0.16	0.11	0.15	0.13	0.11	0.26
Social support										−0.10	0.08	0.22	−0.08	0.08	0.33
Stigma													0.40	0.17	<0.05*
*R* ^2^	0.08			0.21			0.21			0.22			0.24		

All participants were included in the analysis regardless of Social Phobia Inventory score. ref., reference. *P < 0.05, **P < 0.01.

### Social anxiety subcomponents associated with stigma in youths

Table 4 displays the results of a regression analysis predicting stigma scores from the three subscales of social anxiety. Among the subscales, only avoidance was significantly associated with stigma (*β* = 0.17, s.e. = 0.07, *P <* 0.01), indicating that youths who engage in greater social avoidance tend to report higher levels of internalised stigma. Fear (*β* = 0.09, *P* = 0.30) and physical symptoms (*β* = 0.05, *P* = 0.54) were not significant predictors. These findings suggest that avoidance behaviours may play a unique role in reinforcing stigma among socially anxious youths.

**Table 4:** Hierarchical regression predicting stigma score from social anxiety subscales (*N* = 255)

Social Phobia Inventory subscales	*β* (estimate)	s.e.	*t*-value	*P*-value
Fear	0.09387	0.09013	1.04	0.30
Avoidance	0.17149	0.06774	2.53	<0.01^**^
Physical symptoms	0.05380	0.08781	0.61	0.54

** *P* < 0.01.

## Discussion

Both study objectives were met: the post-COVID prevalence and severity of youth social anxiety were quantified (69.55% at least mild, 20% severe), and an integrated model showed that depressive symptoms and internalised stigma, rather than sleep quality or social support, were the principal predictors of anxiety severity. Given the high prevalence of social anxiety symptoms among youth following the COVID-19 pandemic, early identification and timely intervention are critical to preventing long-term psychological and functional impairment. Reducing the stigma associated with social anxiety may promote earlier help-seeking and reduce the risk of comorbid conditions such as depression. In addressing these challenges, emerging technologies, particularly virtual reality-based interventions, may be especially engaging for younger populations.

### Demographic differences in youths

Female participants reported higher social anxiety than males, consistent with prior research.^25^ Biologically, as oestradiol levels rise and fall during the menstrual cycle, the brain’s response to fear changes. When levels are low, fear memories persist more readily and are harder to calm, so social situations can feel much more threatening.^26^ Psychologically, females engage more than males in rumination and post-event self-evaluation, cognitive styles that prolong negative affect and amplify internalising distress, thereby sustaining social anxiety.^27^

Age differences were also notable: high school students (<18 years) exhibited the highest levels of social anxiety, followed by postgraduates, with undergraduate students reporting the lowest levels. Several developmental, contextual and role-transition factors can explain the U-shaped age gradient found in this study. First, mid-adolescence is a peak period for social-evaluative concern: puberty-related changes in self-consciousness, heightened peer salience and high-stakes examinations intensify fear of negative evaluation and avoidance learning, producing the highest rates of social anxiety in secondary school samples.^28^ Second, the dip during undergraduate years may reflect a relatively supportive milieu: universities typically offer greater autonomy, diverse peer networks and structured extracurricular activities that bolster perceived belonging, buffering social anxiety.^29^ Finally, social anxiety rises again in postgraduate study because reintegration into competitive academic roles coincides with supervisor dependence and uncertain career prospects; these stressors erode self-efficacy and reactivate avoidance cycles.^30^ Together, these developmental and environmental shifts map onto the observed U-curve, with adolescent vulnerability, undergraduate reprieve and postgraduate resurgence.

Non-smokers had higher social anxiety (Table 1), but smoking negatively predicted in regression analyses (Table 3). One possible explanation is that socially anxious youths often avoid settings where smoking takes place, whereas more outgoing peers may gravitate towards them. Evidence from a 12-year Finnish twin study (*N* = 1906) showed that adolescents rated high on social anxiety had lower cigarette use and fewer nicotine-dependence symptoms, probably because they avoided smoking-friendly gatherings (e.g. parties).^31^ Consistent with longitudinal social network evidence, youths who reported more close friends also admitted lower social anxiety, suggesting that broader peer networks provide a buffer against anxious distress.^32^ In contrast, we found no link between social anxiety and either BMI or household income. The pattern mirrors a United Arab Emirates University survey (*N* = 553) that reported no correlation between BMI categories and social anxiety scores, and also the population-based Young-HUNT3 study of Norwegian adolescents (*N* = 6600), in which family income strata did not differentiate full, subclinical or performance-only social anxiety presentations (*χ* ^2^ = 2.52, *P* = 0.28).^33^

### Post-pandemic ‘perfect storm’ drives a surge in youth social anxiety

The findings in Table 2 reveal a strikingly high prevalence of social anxiety symptoms among youths, with 69.55% of participants reporting at least mild levels of social anxiety, substantially higher than those reported in pre-pandemic studies. Previous epidemiological data typically indicated that SAD affected approximately 36% of the youth population globally.^3^ The sharp increase observed in this study may reflect the lingering fallout of COVID-19 containment measures, such as lockdowns, remote learning and mask-wearing, which together created a ‘perfect storm’ that heightened youths’ vulnerability to social anxiety.

First, prolonged stay-at-home orders sharply curtailed everyday peer interactions (e.g. classroom chatter, lunchtime conversations, extracurricular activities) that typically provide repeated exposure and gradual mastery of social cues. Without these low-stakes encounters, many youths experienced a kind of ‘deconditioning’: the longer they remained isolated, the more novel and threatening ordinary face-to-face situations felt when restrictions eased.^34^ Thompson et al^35^ documented this trajectory, showing that mean social interaction anxiety scale scores rose significantly during lockdown and remained elevated 3 months after schools had reopened, suggesting that symptoms did not spontaneously remit once isolation ended.

Second, remote learning compounded the problem by shifting virtual academic and social exchanges to screens. Video platforms allow students to mute microphones, turn off cameras or hide behind profile images; these behaviours function as online safety behaviours, mirroring avoidance in vivo and preventing disconfirmation of negative social beliefs.^36^ Consequently, socially anxious students may have received fewer corrective experiences (e.g. realising that minor mistakes are usually tolerated), reinforcing catastrophic predictions about being judged. A Finnish nationwide survey (*N* = 150 000 adolescents) showed that ≥2 months of distance education was associated with a higher risk of clinically relevant social anxiety, even after adjusting for general anxiety and depression.^37^

Finally, mask-wearing produced a subtler, bidirectional effect. On the one hand, masks can act as a portable safety cue that temporarily buffers self-conscious youths from perceived scrutiny; on the other, they obscure facial expressions and muffle speech, increasing ambiguity in social exchanges.^16^ Ambiguous cues are interpreted more negatively by socially anxious individuals, heightening fears of being misunderstood or negatively evaluated.^38^ Moreover, when mandates were lifted, removing the mask required relinquishing this protective barrier, which many youths described as feeling overexposed, leading to spikes in avoidance and worry about appearance or blushing.^15^ In a sample of 223 Chinese university students, higher levels of social anxiety significantly predicted a stronger intention to keep wearing masks after mandates were lifted, indicating that face coverings served as both an impression-management aid and an avoidance mechanism.^16^

These findings offer updated evidence on the severity and scope of social anxiety among youth in the post-pandemic era, underscoring an urgent need for targeted mental health interventions and prevention strategies adapted to this new context. The challenges in early identification have increased due to factors such as the ‘new normal’, including digital learning, prolonged mask-wearing and the shift towards online socialisation, which can obscure typical warning signs. Existing assessment tools and diagnostic methods may no longer fully capture the nuanced presentation of social anxiety in this era, highlighting the need for updated approaches. Consequently, there is a pressing need to develop tailored prevention and treatment strategies that address the unique experiences of youths under these evolving conditions.

### Depression as a robust correlate of social anxiety severity

The regression analysis confirmed that depression is closely intertwined with social anxiety severity. Even when controlling for other factors, depression remained a significant predictor in this study. This aligns with literature showing high comorbidity and interdependence between social anxiety and depression in youth.^9^ Youths with social anxiety often develop depression, possibly due to the chronic stress of social fear and resultant isolation. Conversely, youths with depression may withdraw from social activities and develop secondary social anxiety. Although causality cannot be determined from the current data, the findings reinforce the importance of screening for depression in socially anxious youth. At the same time, social anxiety should be recognised as a potential early warning sign in the identification of youth at risk of depression.

### Why expected buffers failed to predict social anxiety severity

Although sleep disturbances are commonly reported among anxious individuals and are theorised to have a bidirectional relationship with anxiety, the present study found that, after accounting for depression, sleep was not an independent predictor of social anxiety severity. This suggests that the association between poor sleep and social anxiety may be confounded or mediated by depressive symptoms, which themselves can disrupt sleep quality.^11^ Similarly, perceived social support did not independently predict lower social anxiety in the regression model, despite theoretical expectations that it would function as a protective factor. There are two possible explanations for this: first, measurement limitations. Researchers have argued that brief global support scales may not capture the kinds of support most relevant to socially anxious youths (e.g. coaching for challenging conversations, emotion-focused validation after embarrassing moments). The present study likewise relied on a broad social support index, which may have lacked the granularity needed to detect these subtle but important resources. Qualitative work by Nickerson and Nagle^39^ echoes this concern: socially anxious adolescents report valuing highly specific guidance, such as feedback on initiating conversation, yet rarely endorse such needs on general support checklists.^39^ Second, cultural filtering of support perceptions: East Asian youths often downplay personal needs to maintain group harmony; support may be re-coded as unwanted attention, especially in socially anxious individuals who already filter social cues negatively. As a result, youths may subjectively discount otherwise supportive environments, and this tendency is further intensified by the negative cognitive biases characteristic of social anxiety.

### Stigma: a key driver of youth social anxiety

The study found that stigma is also a significant predictor of anxiety severity. This suggests that youths who feel ashamed of their anxiety, or feel that others would judge them for it, tend to have more severe symptoms. This could operate in several ways. Stigma might worsen social anxiety by adding an extra layer of anxiety about the anxiety: for instance, being afraid that one’s anxious behaviours (like trembling or avoiding eye contact) will be noticed and mocked. This meta-anxiety can amplify avoidance and fear, thereby raising overall severity.

Additionally, feeling stigmatised can discourage youths from seeking help or opening up about their fears, potentially prolonging or intensifying social anxiety symptoms. This challenge is particularly pronounced in late adolescence and early adulthood, a developmental stage marked by heightened sensitivity to social evaluation and a still-forming sense of self-worth. Intervention efforts should aim to reduce stigma – for example, through anti-stigma campaigns and anonymous support services.

To break the stigma–anxiety spiral highlighted in this study, two policy moves are paramount. First, mandate annual, school-wide mental health screenings in all lower and upper secondary schools, using brief validated tools for social anxiety, depression and stigma attitudes; early detection will bring hidden cases into view and link students to counselling or digital self-help resources. Second, launch youth-centred anti-stigma campaigns on platforms popular with adolescents, such as BiliBili, WeChat Channels and Douyin, that normalise everyday anxiety, share recovery stories and portray help-seeking as a mark of strength.

### Avoidance as a key mechanism of internalised stigma

A notable finding of this study is that, among the dimensions of social anxiety, avoidance behaviour uniquely correlates with internalised stigma whereas fear and physical symptoms do not. This suggests that avoidance may play a pivotal role in how individuals with social anxiety internalise stigma. Avoidance behaviours, often employed to mitigate immediate anxiety, can inadvertently reinforce negative self-perceptions and social stereotypes, thereby perpetuating internalised stigma.^40^ This cycle not only maintains the severity of social anxiety but also hinders individuals from seeking help due to the fear of judgement.^41^ Clinically, these findings underscore the importance of targeting avoidance behaviours in therapeutic interventions, such as behavioural therapy and virtual reality-based exposure, to reduce social anxiety and associated functional impairments. By addressing avoidance, not only can the cycle of anxiety be disrupted, but the internalised stigma associated with social anxiety can also be alleviated. This dual approach may enhance treatment efficacy and encourage individuals to seek the support they need.

### Recommendations

Clinically, these results underscore the importance of targeting avoidance with evidence-based techniques that also reduce self-stigma. Two options stand out: (a) cognitive–behavioural therapy with graded exposure, which pairs stepwise social challenges with cognitive restructuring to disconfirm catastrophic beliefs, and has consistently strong effects in youth; and (b) virtual reality exposure therapy, which offers realistic yet controllable social simulations and achieves treatment gains similar to in vivo exposure, while also being highly acceptable to youths who may refuse face-to-face exercises. This study highlights the urgent need to bridge the widening gap between adolescents’ evolving mental health needs and outdated support systems – aptly captured by the metaphor, ‘The feet have grown, but the shoes have not changed size’.

### Limitations

Despite its strengths, this study has several limitations. First, the online recruitment method may have limited sample representativeness, introducing selection bias toward tech-savvy or urban youth. Second, another key limitation is the reliance on self-report instruments, which are susceptible to social desirability and recall biases, particularly for sensitive constructs such as stigma. Future studies should incorporate multi-method assessments, including clinician-rated interviews and behavioural tasks, to cross-validate self-reports. Third, the modest *R* ^2^ value of 0.24 in the final model indicates that, while depression and stigma are important predictors of social anxiety, a substantial proportion of variance remains unexplained. This suggests that other unmeasured factors, such as parenting style, peer bullying, family functioning or academic stress, may also play meaningful roles. Future research should incorporate these additional psychosocial and contextual variables to build a more comprehensive predictive model.

## Supporting information

Wang et al. supplementary material Wang et al. supplementary material

## Data Availability

The data that support the findings of this study are available from the corresponding author, Y.-Z.W., upon reasonable request.

## Supplementary material

The supplementary material is available online at https://doi.org/10.1192/bjo.2026.10980
