# Supplementary material for: Prevalence, comorbidity and predictors of social anxiety severity among Chinese youth in the post-COVID-19 era
Source: BJPsych Open. 2026 Feb 26;12(2):e74. doi: 10.1192/bjo.2026.10980 (PMC12963837; doi:10.1192/bjo.2026.10980)
Supplement: Wang et al. supplementary material [file S2056472426109806sup001.docx]

Sensitive analysis 1. Hierarchical regression model on youths with social anxiety (N=386).

| Variable | Model 1 | | | Model 2 | | | Model 3 | | | Model 4 | | | Model 5 | | |
| --- | --- | --- | --- | --- | --- | --- | --- | --- | --- | --- | --- | --- | --- | --- | --- |
|  | *β* | *SE* | *p*-Value | *β* | *SE* | *p*-Value | *β* | *SE* | *p*-Value | *β* | *SE* | *p*-Value | *β* | *SE* | *p*-Value |
| Intercept | 46.88 | *4.87* | <.01** | 47.15 | *5.03* | <.01** | 46.17 | *5.05* | <.01** | 51.05 | *6.74* | <.01** | 41.10 | *7.85* | <.01** |
| Male ref. female | -0.61 | *1.42* | 0.68 | -1.01 | *1.48* | 0.50 | -0.89 | *1.47* | 0.54 | -0.68 | *1.49* | 0.65 | -0.49 | 1.47 | 0.74 |
| Age | -2.84 | *1.27* | <.05* | -1.10 | *1.18* | 0.35 | -1.25 | *1.18* | 0.29 | -1.18 | *1.19* | 0.32 | -1.02 | 1.18 | 0.39 |
| Smoking | 0.11 | *2.49* | 0.96 | -3.78 | *2.36* | 0.11 | -3.65 | *2.35* | 0.12 | -4.12 | *2.39* | 0.09 | -3.85 | 2.37 | 0.11 |
| Friends can rely on | -3.67 | *0.95* | <.01** | -3.48 | *1.02* | <.01** | -3.36 | *1.02* | <.01** | -3.14 | *1.04* | <.01** | -3.01 | 1.03 | <.01** |
| Depression |  |  |  | 0.53 | 0.10 | <.01** | 0.41 | 0.12 | <.01** | 0.39 | 0.12 | <.01** | 0.31 | 0.13 | < .05*** |
| Sleeping |  |  |  |  |  |  | 0.18 | 0.11 | 0.10 | 0.17 | 0.11 | 0.14 | 0.13 | 0.11 | 0.25 |
| Social support |  |  |  |  |  |  |  |  |  | -0.08 | 0.08 | 0.28 | -0.06 | 0.08 | 0.40 |
| Stigma |  |  |  |  |  |  |  |  |  |  |  |  | 0.40 | 0.17 | < .05*** |
| *R* square | 0.05 | | | 0.20 | | | 0.21 | | | 0.21 | | | 0.23 | | |

*Note*: SPIN ≥21participants only (at least mild SA). **p* < .05. ***p* < .01.

Sensitive analysis 2. Hierarchical regression model on youths with social anxiety (N=255).

| Variable | Model 1 | | | Model 2 | | | Model 3 | | | Model 4 | | | Model 5 | | |
| --- | --- | --- | --- | --- | --- | --- | --- | --- | --- | --- | --- | --- | --- | --- | --- |
|  | *β* | *SE* | *p*-Value | *β* | *SE* | *p*-Value | *β* | *SE* | *p*-Value | *β* | *SE* | *p*-Value | *β* | *SE* | *p*-Value |
| Intercept | 38.70 | *5.07* | <.01** | 48.68 | *5.09* | <.01** | 47.74 | *5.11* | <.01** | 53.32 | *6.80* | <.01** | 43.36 | *7.96* | <.01** |
| Male ref. female | 3.75 | *1.42* | <.01** | -0.82 | *1.50* | 0.58 | -0.70 | *1.49* | 0.64 | -0.47 | *1.50* | 0.76 | -0.30 | 1.49 | 0.84 |
| Age | -3.18 | *1.47* | <.05* | -1.78 | *1.18* | 0.14 | -1.92 | *1.18* | 0.11 | -1.82 | *1.19* | 0.12 | -1.69 | 1.18 | 0.15 |
| Smoking | -3.74 | *2.28* | 0.10 | -4.60 | *2.33* | <.05* | -4.53 | *2.33* | <.05* | -5.01 | *2.35* | <.05* | -4.63 | 2.34 | <.05* |
| Friends can rely on | -4.67 | *0.95* | <.01** | -3.40 | *1.04* | <.01** | -3.28 | *1.04* | <.01** | -3.02 | *1.06* | <.01** | -2.89 | 1.05 | <.01** |
| Depression |  |  |  | 0.54 | 0.10 | <.01** | 0.41 | 0.12 | <.01** | 0.39 | 0.12 | <.01** | 0.31 | 0.13 | < .05*** |
| Sleeping |  |  |  |  |  |  | 0.18 | 0.11 | 0.11 | 0.16 | 0.11 | 0.15 | 0.13 | 0.11 | 0.26 |
| Social support |  |  |  |  |  |  |  |  |  | -0.10 | 0.08 | 0.22 | -0.08 | 0.08 | 0.33 |
| Stigma |  |  |  |  |  |  |  |  |  |  |  |  | 0.40 | 0.17 | < .05*** |
| *R* square | 0.08 | | | 0.21 | | | 0.21 | | | 0.21 | | | 0.24 | | |

*Note*: SPIN ≥25 participants only. **p* < .05. ***p* < .01.
